# Precise tactile localization on tools in two dimensions

**DOI:** 10.1016/j.isci.2026.115964

**Published:** 2026-05-22

**Authors:** Matthew R. Longo, Rachel Forde, Jonathan Joel, Luke E. Miller, Alessandro Farnè

**Affiliations:** 1 School of Psychological Sciences, Birkbeck, University of London, London, UK; 2 Donders Institute for Brain, Cognition and Behaviour, Radboud University, Nijmegen, the Netherlands; 3 Integrative Multisensory Perception Action & Cognition Team of the Lyon Neuroscience Research Center INSERM U1028, CNRS U5292, University UCBL Lyon 1, Lyon, France

**Keywords:** biological sciences

## Abstract

The ability to localize touch on the skin is a fundamental perceptual skill. Recent results show that this ability extends to hand-held tools, allowing participants to precisely localize along a rod’s length. This tool-based localization may involve repurposing body-based somatosensory mechanisms. It remains unclear whether precise localization is limited to one-dimensional tools aligned with the long axis of the arm. Here, we show that touch can be localized along multiple dimensions of a held object. Participants held a square board while we measured tactile localization performance. Accuracy was well above chance, both overall and for both the proximodistal and medio-lateral axes. Notably, localization was more precise in the medio-lateral axis, reflecting an anisotropy similar to tactile perception on the skin. This anisotropy was defined in a hand-centered reference frame. These results show that wielded objects can be integrated with the somatosensory system, allowing rich tactile perception from tools.

## Highlights

• Touch can be precisely localized along two dimensions of a hand-held tool
• Clear localization found for both proximodistal and medio-lateral axes
• Localization was more precise in medio-lateral axis
• Tool-based localization reflects hand-centered somatosensory processes

## Graphical abstract

**Figure:**
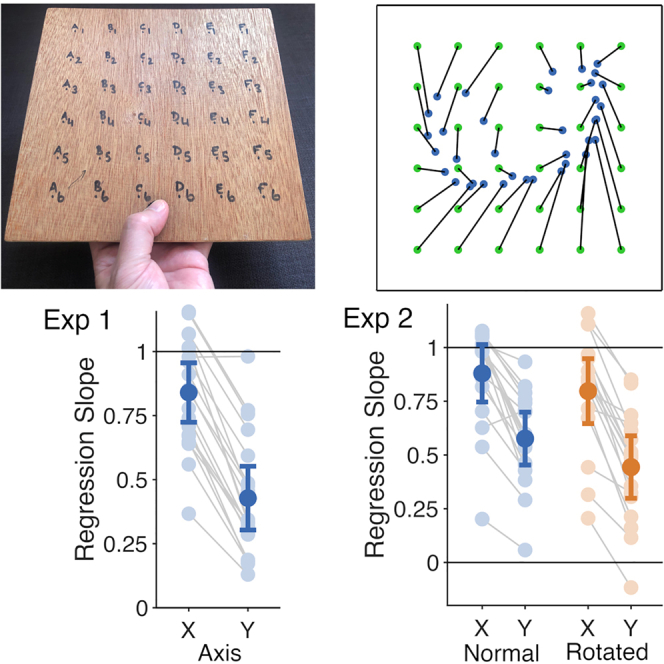

## Introduction

The ability to localize where on the skin a touch occurs is one of the most fundamental perceptual abilities. Neurological studies of patients with brain injury show that tactile localization can be selectively impaired.^1^^,^^2^ And neurocognitive models of high-level somatosensory processing emphasize that it is a process distinct from basic processing of tactile inputs.^3^^,^^4^ The ability to extend tactile localization to wielded tools has been discussed for centuries, most notably in the context of the use of canes by blind people.^5^^,^^6^^,^^7^^,^^8^^,^^9^ As William James^7^ put it, “The draughtsman’s immediate perception seems to be of the point of his pencil, the surgeon’s of the end of his knife, the duellist’s of the tip of his rapier as it plunges through his enemy’s skin” (pp. 37–38). Recent results show, however, that tactile localization is not limited to the tip of a held rod, but extends precisely along the full length of the tool.^10^^,^^11^^,^^12^ This tool-extended localization may involve repurposing body-based somatosensory mechanisms.

One notable feature of these studies is that they involve localization along the length of a rod, which is essentially one-dimensional and oriented with the long axis of the arm itself. It is not known whether this precise tactile localization ability generalizes to multiple axes of a held object. Notably, a series of studies by Ujitoko and colleagues shows that tactile localization on a two-dimensional tabletop that the hand is resting on is extremely poor.^13^^,^^14^^,^^15^ While these results show that tactile localization in 2-D space is not trivial, localization ability in a situation where the hand merely rests on a surface may not generalize to the case where the surface is actively held.

In this study, we tested this hypothesis, measuring tactile localization ability in 2-D space on a square wooden board held in the left hand. We used an established task which has been widely used to measure tactile localization both on the body^16^ and on tools.^10^^,^^11^^,^^12^ If tools are tightly integrated with the somatosensory system, localization performance should generalize to multiple axes of tools just as stimuli can be localized in multiple body axes.

## Results

### Touch can be localized on tools in both the proximodistal and medio-lateral axes

**Figure 1:**
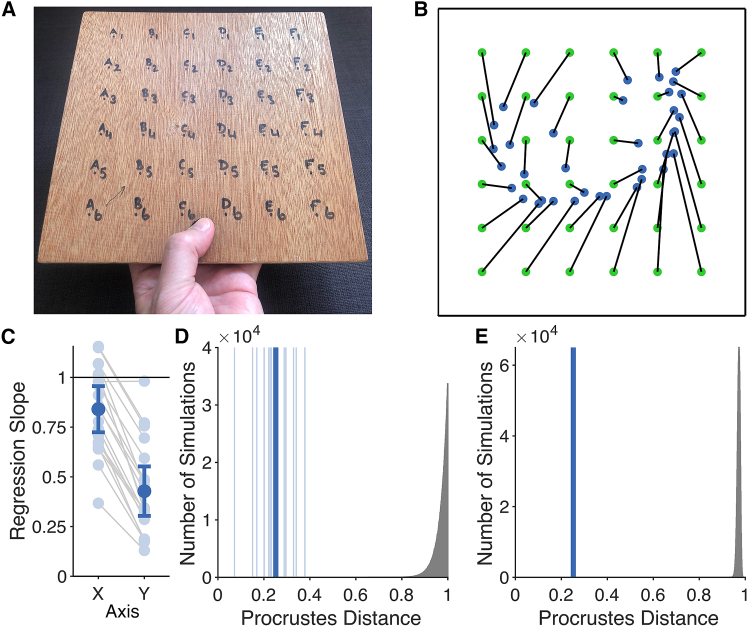
Experimental paradigm and results from Experiment 1 (A) The board that participants held (background) with 36 stimulus locations marked. (B) Grand average localization performance in Experiment 1 showing actual locations (green) and mean judged locations (blue). (C) Slopes regressing judged location on actual location separately for the medio-lateral (X) axis and the proximodistal (Y) axis. If participants are unable to localize touch, regression slopes should, on average, equal 0. Optimal performance would yield a slope of 1. Error bars are 95% CIs. (D and E) Procrustes distances comparing perceptual maps with actual stimulus locations. Thin vertical lines are individual participants, while the thick line is the grand mean. The gray histogram shows a null distribution generated from simulations of single participants (D) or from a sample of 20 participants (E).

Participants held a square wooden board in their left hand, as shown in Figure 1A. On each trial, one of the 36 locations on the board was tapped and participants indicated the perceived location of touch by clicking a picture of the board on a monitor (STAR Methods). The resulting maps are shown in Figure 1B. The similarity of perceptual maps of judged location to actual layout was quantified by the Procrustes distance between them, which was significantly smaller than chance for all participants (results in Figure 1D).

Performance was also quantified in each axis by regressing judged location on actual location (Figure 1C). In the proximodistal axis, slopes were significantly higher than 0 (*M*: 0.428, *SD*: 0.229), *t*(19) = 8.37, *p* < 0.0001, *d* = 1.87, consistent with results using a one-dimensional rod.^10^^,^^17^ More critically, slopes were also higher than 0 in the medio-lateral axis (*M*: 0.840, *SD*: 0.213), *t*(19) = 17.61, *p* < 0.0001, *d* = 3.94. This shows that localization on held objects is not specific to the proximodistal dimension, but occurs precisely in two-dimensional space.

### Precision of localization is higher in the medio-lateral than the proximodistal axis

We next compared regression slopes in the medio-lateral and proximodistal orientations. Slopes were strongly correlated in the two orientations, *r*(18) = 0.770, *p* < 0.0001, suggesting that both reflect a common perceptual ability which differs systematically across individuals. Notably, however, regression slopes were even higher in the medio-lateral than in the proximodistal hand axis, *t*(19) = 12.24, *p* < 0.0001, *d*_*z*_ = 2.74. To further investigate this anisotropy, we calculated the variable error across trials. For each participant, we calculated the standard deviation of judgments separately for each of the 36 locations and separately for the x- and y-coordinates. Variable error was significantly smaller in the medio-lateral than in the proximodistal axis (0.121 vs. 0.137 Bookstein units), *t*(19) = 2.41, *p* < 0.05, *d*_*z*_ = 0.539. This is consistent with previous studies that have found reduced variable error in the medio-lateral axis of the hand itself.^18^^,^^19^

### Localization occurs in a hand-centered frame of reference

**Figure 2:**
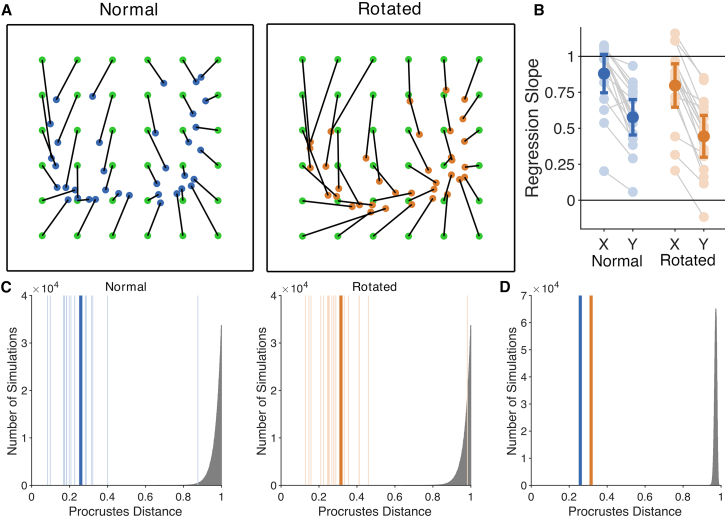
Results from Experiment 2, manipulating arm posture (A) Grand average localization performance in the normal (*left*) and rotated (*right*) postures. (B) Regression slopes in both axes for the two postures. Error bars are 95% CIs. (C and D) Procrustes distances comparing perceptual maps with actual stimulus locations for the normal posture (blue) and the rotated posture (orange). Thin vertical lines are individual participants, while the thick line is the grand mean. The gray histogram shows a null distribution generated from simulations of single participants (C) or from a sample of 18 participants (D).

If this localization results from the integration of the tool with somatosensory hand representations, localization on the tool should occur in a hand-centered reference frame. Experiment 2 investigated this issue, taking advantage of the fact that the precision of localization differed between the two axes of the board, comparing conditions in which the arm was held straight ahead (*normal* posture) or in which the forearm was rotated 90° so that the hand and board were pointing to the participant’s right (*rotated* posture). Results from the normal posture replicated Experiment 1 (Figure 2), with Procrustes distances comparing actual and perceived maps smaller than chance for 17 of 18 participants. Slopes were greater than 0 in both the proximodistal axis (*M*: 0.576, *SD*: 0.212), *t*(17) = 11.52, *p* < 0.0001, *d* = 2.71, and the medio-lateral axis (*M*: 0.880, *SD*: 0.231), *t*(17) = 16.18, *p* < 0.0001, *d* = 3.81, which were again strongly correlated, *r*(16) = 0.708, *p* < 0.001. Again, slopes were significantly greater in the medio-lateral than the proximodistal axis, *t*(17) = 7.57, *d*_*z*_ = 1.79.

Results in the rotated posture were nearly identical, with Procrustes distances smaller than chance for 17 of 18 participants. Slopes were greater than 0 in both the proximodistal axis (*M*: 0.444, *SD*: 0.251), *t*(17) = 7.50, *p* < 0.0001, *d* = 1.77, and the medio-lateral axis (*M*: 0.797, *SD*: 0.261), *t*(17) = 12.98, *p* < 0.0001, *d* = 3.06, which were strongly correlated, *r*(16) = 0.813, *p* < 0.0001. Slopes were significantly greater in the medio-lateral than the proximodistal axis, *t*(17) = 9.57, *d*_*z*_ = 2.26. The highly similar results in the two postures indicate that localization occurs in a hand-centered reference frame. This suggests that the somatosensory mechanisms which ordinarily serve tactile perception on the skin are re-purposed to support tactile localization on the tool.

Finally, analyses of standard deviations of judgments showed that variable error was significantly smaller in the medio-lateral than the proximodistal axis of the body, both in the normal posture (0.110 vs. 0.136), *t*(17) = 2.87, *p* < 0.02, *d*_*z*_ = 0.676, and in the rotated posture (0.127 vs. 0.150), *t*(17) = 3.12, *p* < 0.01, *d*_*z*_ = 0.735, again replicating the pattern of results from Experiment 1.

## Discussion

Our results clearly replicate previous studies showing precise tactile localization along the proximodistal axis of a long rod,^10^^,^^11^^,^^12^ similar to a cane. Crucially, we demonstrate robust localization in the orthogonal medio-lateral axis. Indeed, performance was even higher in the medio-lateral axis than in the proximodistal axis, which may relate to known anisotropies in tactile spatial acuity^20^ and tactile distance perception.^21^ This shows that the ability to localize touch on hand-held objects is not specific to a single axis of a long rod, but reflects a more general spatial ability to incorporate them into the somatosensory system when they are used for sensing the environment. In this sense, our results parallel and extend recent findings of precise tactile perception of stimuli applied to fingernails^22^^,^^23^ and hairs.^24^

The present results contrast with results showing poor localization performance of taps applied to a tabletop on which the hand rests.^13^^,^^14^^,^^15^ This dissociation suggests that active holding may activate embodiment mechanisms that extract touch location in a way that mere contact does not. Indeed, the precision of tactile localization is higher still when a rod is actively lowered by the participant onto a stimulus.^10^ Computational modeling showed that tactile localization on a one-dimensional rod could be supported by populations of vibration-sensitive Pacinian mechanoreceptors.^10^ The same mechanism may operate here. It is likely that proprioceptive signals related to finger movements and postural adjustments also contribute to performance in the present task. This possibility is consistent with the finding of Miller and colleagues^10^ that localization performance is even higher when the tool is actively moved by the participant. In this sense, the localization ability described here should be thought of as haptic, rather than purely tactile.

The precision of localization was higher in the medio-lateral axis than in the proximodistal axis. This mirrors anisotropies in tactile spatial perception of touch on the hand, for example, in tactile spatial acuity^20^^,^^25^^,^^26^ and tactile distance perception.^21^^,^^27^ Thus, wielding a tool projects not only the sensory abilities of the hand onto the tool, but also its characteristic anisotropies and distortions.^28^ This provides further evidence that the mechanisms subserving tactile localization on the skin are repurposed for perception on tools.^10^^,^^17^ It is also possible that the particular task used here, or the particular posture used to hold the board, may have differentially influenced performance in the two axes of the board.

### Limitations of the study

There are several limitations of this study. First, the present findings are based on a single, flat, square, wooden object, and it remains unclear if the results would generalize to tools of different shapes, dimensions, and materials. Second, performance in the present study likely reflects the combined contribution of several sensory input channels, including pressure, vibration, and proprioception, which cannot be dissociated in the present design. Future research could investigate the specific contributions of each of these signals. Third, while the participants held the board, they did not actively wield it, a factor which is known to enhance tactile localization ability.^10^

## Resource availability

### Lead contact

Requests for further information and resources should be directed to and will be fulfilled by the lead contact, Matthew Longo (m.longo@bbk.ac.uk).

### Materials availability

All materials are available on the Open Science Framework (https://osf.io/cgv6n/).

### Data and code availability

Raw data and analysis code are available on the Open Science Framework (https://osf.io/cgv6n/).

This paper does not report original code.

Any additional information required to reanalyse the data reported in this paper is available from the lead contact upon request.

## Acknowledgments

This work was supported by the 10.13039/501100001665Agence Nationale de la Recherche (ANR-24-CE37-5761) ExSoSpace to A.F.

## Author contributions

Conceptualization: M.R.L., R.F., J.J., L.E.M., and A.F.; formal analysis: M.R.L.; investigation: R.F. and J.J.; methodology: M.R.L., R.F., and J.J.; software: M.R.L.; supervision: M.R.L.; visualization: M.R.L.; writing – original draft: M.R.L; writing – reviewing and editing: R.F., J.J., L.E.M, and A.F.

## Declaration of interests

The authors declare no competing interests.

## STAR★Methods

### Key resources table

**Table:** 

REAGENT or RESOURCE	SOURCE	IDENTIFIER
**Software and algorithms**		

MATLAB R2016B	Mathworks	https://uk.mathworks.com/
PsychToolbox	Brainard^29^	http://psychtoolbox.org/

### Experimental model and study Participant Details

#### Experiment 1

Twenty members of the Birkbeck community (12 women, 8 men) between 24 and 61 years of age (*M*: 35.6 years, *SD*: 8.5) participated in Experiment 1. All participants but 3 were right-handed as assessed by the Edinburgh Inventory^30^ (*M*: 66.9, *SD*: 53.1). Participants had normal or corrected-to-normal vision and reported no abnormalities of sensory function. Participants provided written informed consent before participating. All procedures were approved by the School of Psychological Sciences Research Ethics Committee at Birkbeck (approval number: 171887).

#### Experiment 2

An additional 18 individuals (7 women, 11 men) between 19 and 59 years of age (M: 31.8 years, SD: 11.9) participated in Experiment 2. All participants were right-handed (*M*: 85.0, *SD*: 16.8).

### Method details

#### Experiment 1

The participant sat comfortably on a chair in front of a table. They were asked to place their left arm behind a screen with their wrist resting on a cushion. They held a wooden board (23 cm x 23 cm square, 5 mm in height, 164 g in weight). A Velcro disk on top of the board indicated where they should place their thumb. The participant was given the following instructions about how to hold the board:

“To hold the board please place your left thumb on the Velcro circle and support the underside of the board with your four fingertips spread evenly. Please hold the board so your grip is firm enough for it not to fall easily from your hands when tapped, but not so firmly that it has no movement when tapped.”

Thirty-six locations were marked on the board in a 6x6 grid using a black pen. The distance between each mark and between the outside marks and the edge of the board was 3.3 cm.

The experimental paradigm was based on the task developed by Mancini and colleagues^16^ and which has been widely used, including in previous studies of tactile localisation on tools^10^^,^^31^ and on fingernails.^22^ On each trial, a single short tap was applied by the experimenter to one of the locations using their index finger. The experimenter was as consistent as possible in the pressure and duration of the tap. After the tactile stimulus was applied, a brown square appeared on a 24” monitor approximately 40 cm in front of the participant (1600 x 1200 pixels, 75 Hz refresh rate). The brown square was the same physical size as the board and its colour was determined as the average RGB value of the board calculated from a photograph. The participant’s task was to use their right hand to move the mouse cursor (a crosshair icon) to the location on the square corresponding to where they felt the touch. The starting location of the crosshair on the screen was randomised on each trial. The experiment was controlled by a custom MATLAB (MathWorks, Natick, MA) script using the PsychToolbox.^29^

There were 8 blocks of trials, each of which included one trial for each of the 36 stimulus locations in random sequence. There were thus 288 trials in total.

The participant wore headphones playing white noise to mask any potential audio cues from the board being tapped.

#### Experiment 2

Procedures were identical to Experiment 1 except that the participant held the board in two different postures across blocks. In the *normal* posture, the board was held with the hand pointing away from the body, as in Experiment 1. In the *rotated* posture, in contrast, the left forearm was rotated 90° clockwise, so that the hand pointed to the participant’s right.

There were four blocks of trials, two each of the normal and rotated postures. The order of blocks was counterbalanced across participants using an ABBA design. Each block included 2 repetitions of each of the 36 locations. This resulted in 72 trials per block and 288 trials in total.

### Quantification and statistical analysis

#### Experiment 1

The analysis approach was based on that used in our previous studies of tactile localisation on tools^10^^,^^17^ and fingernails.^22^ A first analysis used Procrustes alignment^32^ to superimpose the two-dimensional perceptual maps of judged location onto the actual configuration of the 36 stimulus points. Procrustes alignment translates, scales, and rotates two spatial configurations so that they are aligned as closely as possible without distorting the relative spatial configuration of points. The dissimilarity between the two configurations can then be quantified as the root mean squared value of the distances between homologous points in the two maps after alignment, a value known as the *Procrustes distance*. If the maps align perfectly, the Procrustes distance should be 0.

To test for statistical significance of Procrustes distances, a null distribution was created using one million simulations of random data using a custom MATLAB script. Each participant’s Procrustes distance can thus be assessed for statistical significance by determining what proportion of these simulations generated Procrustes distances smaller than each actual value. A null distribution for the grand average Procrustes distance was created by taking 1 million samples of 20 values from the previously described distribution of simulations, allowing the statistical significance of the grand mean Procrustes distance to be assessed.

A second analysis was based on the regression procedure used by Miller and colleagues^10^^,^^17^ in their studies of tactile localisation on tools. Judged location was regressed on actual location for each participant separately. In the studies of Miller and colleagues, the tool was a one-dimensional rod, so only a single regression analysis was conducted for each participant. In the present study, in contrast, the board was two-dimensional, so separate regression analyses were conducted for the proximal-distal axis (i.e., the y-coordinate of location) and the medio-lateral axis (i.e., the x-coordinate of location), as in our recent study of tactile localisation on fingernails.^22^ If participants have no ability to localise touch, then regression slopes should on average equal 0. In contrast, optimal performance would correspond to a slope of 1. The ability to localise at better than chance levels can therefore be assessed by a one-sample t-test comparing mean regression slope to 0.

The coordinates of actual locations and judged locations were brought into a common reference frame using Bookstein’s^33^ two-point registration method in which two landmarks are defined as points (0,0) and (1,0) of a coordinate system, with the second axis defined orthogonal to the first. These landmarks were the bottom left and bottom right corners of the board, respectively. This means that the resulting x-coordinate indicates location in the medio-lateral axis of the board and the y-coordinate indicates location in the proximo-distal axis.

Trials (0.17%) were excluded from analyses if the location clicked was outside of the square on the monitor (i.e., outside of the board itself).

MATLAB scripts used for data collection and analysis and raw data from both experiments are available at: https://osf.io/cgv6n/

#### Experiment 2

The analysis was identical to Experiment 1 except that separate analyses were conducted for the two postures. In addition, the null distribution for the grand-mean Procrustes distances was based on simulations selecting samples of 18 values (rather than 20, as in Experiment 1) to match the sample size of this experiment.

A total of 0.44% of trials were excluded due to the participant clicking outside of the square.
